# The Importance of Understanding Social and Cultural Norms in Delivering Quality Health Care—A Personal Experience Commentary

**DOI:** 10.3390/tropicalmed5010022

**Published:** 2020-02-05

**Authors:** Ahmed S. Latif

**Affiliations:** Public Health Physician, Formerly Professor of Medicine and Dean of College of Health Sciences, University of Zimbabwe, Harare, Zimbabwe; aslatif@gmail.com

**Keywords:** social determinants of health, cultural safety in health service delivery, cultural competency

## Abstract

The objectives of this paper are to provide a review of the author’s personal experiences working in culturally diverse environments and to emphasize the importance of recognizing the social determinants of health. While some determinants of health are modifiable others are not, in addition it is emphasized that cultural safety in delivering health care is crucial if services provided are to be appropriate and acceptable to health care seekers. Cultural sensitivity is needed if one is to make a change in health outcomes in culturally diverse environments. The development and delivery of culturally safe services is more acceptable to community members and is important if a difference is to be made in health inequities. Training in delivering culturally safe services should include both theoretical and practical components. Practical training should be conducted under supervision in remote settings so that trainees appreciate what their clients experience on a daily basis. Culturally “unsafe” clinical service has serious adverse effects. This commentary discusses the above factors and provides example cases from the author’s own career of where such factors have affected the health of individuals or groups.

## Key Messages:

- There are a number of social determinants of health, some of these are modifiable while others such genetic make-up, gender and age are not.
- All health workers should receive education on the specific needs of their patients based on their culture, and, should understand that in a multi-cultural society, approaches to health delivery vary when dealing with patients differing cultures.
- In order for health care services to be effective they need to be both appropriate and acceptable and should be delivered by skilled and competent health care workers.
- Cultural awareness and cultural competency are required for cultural safety.
- Training for delivering culturally safe health care should include both theoretical and practical components.
- Not providing culturally safe services may result in serious adverse outcomes.

## 1. Introduction

In the early days of our own training, more than 50 years ago, we were made to learn of these factors. A number of texts by the very distinguished and respected Professor Michael Gelfand [1,2,3] were available. We were taught that “unless you approach your patients with understanding you will fail to win them over and as a result you will often be unable to cure them.” This is quoted in a review of the publication in the Journal of the American Medical Association in 1964 [4]. As medical students, we took part in the “Medical Anthropology” described in the book, and, as part of this we visited remote communities in Zimbabwe (then Rhodesia) and learnt how traditional practitioners delivered health care to clients that consulted with them for their needs. More recent publications are also available, and a paper by Professor John Goldsmid is referenced here [5]. Professor Goldsmid emphasizes that human behaviour and cultural practices can have a profound effect on the range and prevalence of diseases suffered by communities and that human behaviour is the forgotten factor in disease prevalence and transmission.

My training in medicine was in Zimbabwe. After graduation in 1969, I worked as an intern, senior house officer, and a general practitioner before specializing in internal medicine and sexual health. I worked as a medical officer in the Harare City Health Department where I introduced the concept of the syndromic management of sexually transmitted infections. After specializing I joined the Department of Medicine of the University of Zimbabwe Medical School where I was involved in teaching undergraduates and postgraduates and conducting research and other academic activities. During this time I also worked as a consultant for the World Health Organization and was able to visit numerous countries where I was involved in training doctors and health staff and develop and set up programs for the management and control of sexually transmitted infections (STIs) and Human Immunodeficiency Virus (HIV) infection. I became Professor of Medicine at the University of Zimbabwe College of Medicine and later became Dean of the College of Medicine. Following this I took up an appointment as Public Health Medical Officer with the Department of Health of the Northern Territory in Australia and was based in Alice Springs, developing and implementing STI control activities. This provided me the opportunity to work Indigenous Aboriginal Communities in the Northern Territory of Australia and to learn the customs and needs of the local communities. In 2009, I accepted the post of Medical Director of an Aboriginal Community Controlled Health Organization in the Northern Territory, providing comprehensive primary health care and population health.

In order to improve the health status of an individual, the health status of the population in which the individual lives and works needs to be improved. Population health is defined as health outcomes of a group of individuals. Population health focuses on specific populations looking at a broad range of factors that affect health. These factors are known as social determinants of health. The World Health Organization lists the following factors that influence the health and well-being of individuals [6]:

- Income and social status
- Level of education
- Physical environment—including safe housing, clean air, safe water, healthy workplaces and healthy communities
- Social support networks—including support from family, friends and community, and, effect of individual behaviour
- Culture, customs and traditions
- Genetics—having a predisposition for some diseases and abnormalities
- Access to health services
- Gender of the individual—some illnesses are more frequent in males than females and vice versa

It is important that all trainees in the health service delivery area are taught that the health of their clients is affected by these factors and that without addressing these aspects the client’s health may not improve or their problem will re-occur.

The importance of alternative medical systems cannot be over-emphasized. Depending on the region, country or continent, the public will seek care in alternative health systems. Hence the need to understand both what exists, and, what the health seeking behaviour of individuals is. In some countries in Southern Africa, the traditional healers play an important role in managing clients with chronic conditions and of conditions which suddenly develop without an obvious cause. An example of this is when the epidemic of HIV infection and AIDS started especially at the time (early in epidemic) when the cause of AIDS had not been established and no curative treatment was available. Illness may be “blamed” on misfortune or witchcraft and bad spirits, and hence clients would seek help in the alternative sector [7]. In many societies, the concept of disease origin is not completely understood by some of the populace, an example of this being the aetiology of disease [8]. The concept of the role of “invisible” pathogens and organisms, treatable or not, is not fully understood and requires a great deal of general education.

**Example Case 1**

Early during the course of the outbreak of HIV infection and AIDS in Zimbabwe, before the etiologic agent, Human T Cell Lymphotropic Virus Type 3 (HTLV3 later the nomenclature changed and the etiologic agent was called the human immunodeficiency virus (HIV) was identified, we were faced with patients presenting with symptoms and signs of AIDS-associated conditions such as generalized lymphadenopathy, wasting, opportunistic infections and generalized aggressive Kaposi Sarcoma. In informing patients that there was no known cause for the condition, we inadvertently encouraged patients to seek care in the alternate sector. Later, when the etiologic agent was identified and patients were told that there was no known cure for the infection made patients more steadfast in seeking the help of traditional practitioners known as Ngangas. The services of the traditional practitioners were acceptable to care seekers who felt assured that they could be cured as they had always consulted with them from their early childhood days. Unfortunately, the Ngangas did not have a cure for this devastating infection which took the lives of thousands of infected persons. In the early days of the HIV epidemic, we had extensive discussions with traditional healers in Zimbabwe and even encouraged them to take part in some well-designed research activities.

The determinants of health are numerous. Some of these are non-modifiable, while others may be altered to the beneficial effects of individuals and communities. Non-modifiable determinants include age, gender, genetic make-up and family history. These are ingrained factors and are not modifiable. Modifiable determinants of health include: education, employment, financial income and social status, physical environment both at work and at leisure, housing and social environment, healthy childhood development, culture and personal and community practices, and availability of health services and social support [9,10,11].

## 2. Population Health and Public Health

Population health focusses on specific communities and populations to determine factors that influence health. These factors include the social determinants of health listed above. While population health focuses on specific populations or communities to improve the social and economic well-being as a whole, public health is the organized effort to keep persons and communities healthy and disease-free and to prevent injury, illness and premature death [12]. Constituents of public health include health protection, health surveillance, disease prevention, injury prevention, and health promotion. Population health and public health are closely related and work together hand-in-hand.

In providing health care it is important to understand that health services should at all times be accessible as well as acceptable.

It should however be noted that health care systems alone cannot improve population health without reducing the population’s health and social inequities. A number of studies have shown that population health can improve if social needs are addressed and social conditions are improved [13].

The health of the individual and therefore of the community health is influenced by a number of complex factors that include: provision of acceptable, accessible and appropriate health care, individual and community health behaviours, physical and social environment, socioeconomic status, and public policy. These are all inter-related:

- Risky behaviours such as unhealthy diet and physical inactivity are linked with chronic diseases, but these risk factors depend on the community environment as people can only choose healthy options if these are available, and if the choices they make are safe.
- Socioeconomic status has a great influence on health as having the resources allows people to afford medical care, nutritious foods, and decent housing.

Public policy influences all of these aspects as it addresses education, employment and inequities, both nationally and locally, in both the private and public sectors.

In developing and providing health care services it is important to keep in mind a number factors including whether the service being provided is appropriate for the community receiving the service, whether the service is accessible, and finally whether the service is acceptable. These aspects need careful consideration and will determine whether the services will be used by the community. Considerations include opening hours for the service, gender distribution of staff providing services, distance of service from where people live, availability of transport, and costs for seeking the service.

## 3. Cultural Awareness

The health care provider’s cultural awareness is their understanding of the differences between themselves and people from other backgrounds, especially relating to differences in values and attitudes [14]. It is important to understand that a lack of awareness can lead to bad or poor decision-making and poor outcomes for persons that we are supposedly providing help. Cultural awareness helps to reduce the chances of making bad decisions and increases the chance of us making appropriate and acceptable decisions.

### Training in Cultural Awareness

It is important that health care providers have an understanding of what may or may not be culturally acceptable to the clients that they are dealing with. A number of different approaches are available in delivering cultural awareness training. Training may be theoretical or practical, however, ideally both these components are necessary in order to ensure that the trainee is well-equipped in delivering high quality, acceptable care. Theoretical training may be used to sensitize trainees in being aware that their clients’ needs and beliefs may be different from what they themselves understand. It trains care providers in having an open mind and to ask their patients the right questions in the most acceptable way. Practical training is necessary to ensure that trainees consult with their patients and deliver care in an appropriate and acceptable way.

A number of training programs are available including face-to-face training, reading, and on-line courses. Some educational institutes have developed questionnaires that can be used when consulting with patients. Health care providers can ask a set of questions in order to determine the patients’ social status and figure out what the role of social determinants may play in their health. The American Academy of Family Practice has developed a screening tool to determine whether the patient has a need for social services such as housing, food security, transportation, and to determine the patient’s personal safety issues [15]. This short questionnaire may be used to determine whether the patient has modifiable social determinants of health. Training should aim to make health care providers competent in delivering culturally appropriate health care [16].

However, short training courses may not be sufficient for the delivery of culturally appropriate and culturally safe health care as shown in the Closing the Gap Report of The Australian Institute of Health and Welfare (AIHW) of the Australian Government published in 2015. This report states that cultural competency improves accessibility and effectiveness of healthcare for Indigenous people, however, in Australia, there is no coherent approach to embedding cultural competence in health services and that there is little evidence of how best to improve culturally competent healthcare delivery to Indigenous Australians [17].

Working in remote communities in the Northern Territory of Australia has been a great learning experience as well as an eye-opener. Firstly because of the remoteness of communities it is always difficult to recruit health staff. And secondly, health staff recruited tend to remain a short while. This requires that training needs to be repeated often. Staff obtained through recruitment agencies may come from all over Australia and even overseas. Recruitment agencies endeavor to train new recruits in cultural competency but of necessity the method of training is the short course type or even simply on-line. This may provide the trainee with an overview but does not provide them with a hands-on type experience. Hence, health organizations recruiting new staff need to develop their own cultural awareness and competency training. This is fine for long-term recruits but as often happens such recruits come in on short contracts. There is, therefore, a need for cultural awareness and competency training to take place during the undergraduate training of students in all disciplines of health.

**Example Case 2**

The lack of cultural awareness by health care providers can lead to non-acceptance of services provided. This is particularly important in providing sexual health care services. In this situation the gender of health care providers should match the gender of care seeker. When providing services for women it is important for the patient to be seen by a female care provider, and similarly when dealing with male clients the provider should be male. In my own experience both in Africa and in Australia, I found that it is better not to raise certain topics when managing patients of the opposite gender. In fact, I realized that some words are considered taboo when uttered by a male during a discussion with a female patient. Being aware of such sensitivity is important if trust is to be gained from your patients. In situations where it is necessary for the patient to be provided the information, I found that by bringing in another female staff member (with the permission of the patient) to talk to the patient in my absence was preferable and more acceptable to the patient. When I was in formal non-clinical meetings with senior executive staff, I was advised by female executive staff members that they would prefer to have discussions on sexual health topics with a female clinician.

Being culturally aware is to have the knowledge and being culturally competent is to practice in a culturally safe manner.

In order for health care services to be effective, health care that is provided needs to be acceptable to the people served. Health care services will be considered acceptable to people if they are delivered in a culturally appropriate manner. The provision of culturally appropriate care requires an understanding of the social life and customs of the population served. In Zimbabwe and many other developing countries in Africa the medical teaching curricula include theoretical and practical training in this field. Medical students are exposed to the structure and function of traditional healing process and to social and cultural norms by having to live and learn in remote communities during their undergraduate training. During such remote placements a group of students would live in the community for periods of 3–4 weeks with their medical school supervisors and become involved in dealing with medical problems that the community has to face. Depending on the stage of the students’ medical training, they are required to provide solutions to real-life or hypothetical problems that a community may face. This form of community orientated problem-based teaching takes place annually addressing different scenarios. In this way students learn to work with leaders and community decision makers including elders and traditional practitioners in dealing with major and minor issues that the community faces. The medical curriculum of the University of Zimbabwe College of Medicine is a comprehensive integrated curriculum which allows students to learn through problem solving exercises. Each year of their undergraduate training, students spend to three weeks in remote communities with supervisors and are exposed to local problems. They will observe and, where possible, assist the local community in dealing with existing problems and help identify causes of such problems as well as how to manage them locally. Medical students are placed in different communities in each of their placements.

With this approach students learn of the social, economic and physical environment that their patients come from and also get an understanding of the person’s individual characteristics and behaviours. Importantly, in addition the student is able to identify the social support networks available to patients locally. It is well understood that greater support from families, friends, and communities is linked to better health. Studying during remote placements allows students to learn the culture, customs, traditions, and the beliefs of the family and community, all of which affect health.

## 4. Cultural Safety

A number of different terms have been used to describe the provision of acceptable and appropriate care for persons belonging to cultures that differ from that of the care provider. While these are processes that lead to the provision of acceptable care, cultural safety is the outcome of these processes. With patient safety in mind, the concept of cultural safety is defined by the Australian Health Practitioner Regulation Authority (AHPRA) as the individual and institutional knowledge, skills, attitudes and competencies needed to deliver optimal health care for Aboriginal and Torres Strait Islander Peoples [18]. The Cultural Safety covers a large number of terms currently used interchangeably, including: cultural awareness, cultural competence, cultural capability and proficiency, cultural respect, cultural security, cultural appropriateness, cultural understanding, and cultural responsiveness. These are all important in leading to the main outcome of cultural safety. In its statement AHPRA states “that patient safety for Aboriginal and Torres Strait Islander Peoples is the norm and that patient safety includes the inextricably linked elements of clinical and cultural safety, and this link must be defined by Aboriginal and Torres Strait Islander Peoples”.

The importance of cultural safety cannot be over-emphasized especially since Australia is composed of a multi-cultural society in which everyone has the right to be treated with respect and dignity and this is crucial when delivering health care. Culturally safe health care delivery is crucial in attempting to close the gap in health outcomes in all communities.

Not providing a culturally safe service may lead to ill health such as low utilization of available services, non-compliance with referrals or prescribed interventions, reluctance in interacting with service providers, anger, and, dissatisfaction with tools and interventions used in the dominant culture [19].

In remote Aboriginal communities it is often stated that persons with chronic diseases are often not compliant with medicines prescribed for their illnesses. However, the reason for non-compliance may well be attributed to the lack of cultural awareness and competence of health practitioners, and this matter should be addressed.

## 5. Conclusions

Individual health is determined by a number of factors. While some of these factors are engrained in the individual and are not modifiable, the majority of determinants are modifiable. As health care providers it is our duty to identify the modifiable risk factors and initiate steps to modify these. Health is affected by the place the patient lives in, where they work, as well as where they are schooled. It is known that by eating well and staying active, not smoking, and seeking care when needed all influence health outcomes. Access to social and economic opportunities and availability of resources as well as availability of clean water and air also affect health and wellbeing [20]. Hence, addressing the social determinants health is important in achieving the goal of closing the gap in health inequities.

Training in cultural awareness and competency may be theoretical or practical. Theoretical training raises cultural awareness and provides guidance on its various different components and how to approach the topic. However, practical training provides the trainee with the experience necessary to provide culturally appropriate care and sensitizes the trainee on the multifactorial nature of the social determinants that the patient is surrounded by. Training in the classroom situation is not sufficient to reach the goals of provision of culturally appropriate care. By learning within the community, trainees are able to appreciate the fuller extent of the root causes of their patients’ ill health, and by understanding these, the care provider is able to “manage” their patient in a complete and comprehensible way. Cultural competency training should be a mandatory requirement for all trainees in every branch of the health field.
